# Water cavitation from ambient to high temperatures

**DOI:** 10.1038/s41598-021-99863-z

**Published:** 2021-10-21

**Authors:** Francesco Magaletti, Mirko Gallo, Carlo Massimo Casciola

**Affiliations:** 1 Advanced Engineering Centre, School of Computing Engineering and Mathematics, University of Brighton, Lewes Road, Brighton, BN2 4GJ UK; 2 Department of Mechanical and Aerospace Engineering-DIMA, Sapienza Universitá di Roma, 00184 Rome, Italy

**Keywords:** Mechanical engineering, Engineering, Physics

## Abstract

Predicting cavitation has proved a formidable task, particularly for water. Despite the experimental difficulty of controlling the sample purity, there is nowadays substantial consensus on the remarkable tensile strength of water, on the order of −120 MPa at ambient conditions. Recent progress significantly advanced our predictive capability which, however, still considerably depends on elaborate fitting procedures based on the input of external data. Here a self-contained model is discussed which is shown able to accurately reproduce cavitation data for water over the most extended range of temperatures for which accurate experiments are available. The computations are based on a diffuse interface model which, as only inputs, requires a reliable equation of state for the bulk free energy and the interfacial tension. A rare event technique, namely the string method, is used to evaluate the free-energy barrier as the base for determining the nucleation rate and the cavitation pressure. The data allow discussing the role of the Tolman length in determining the nucleation barrier, confirming that, when the size of the cavitation nuclei exceed the thickness of the interfacial layer, the Tolman correction effectively improves the predictions of the plain Classical Nucleation Theory.

## Introduction

Experimental evidence reveals that pure water at ambient conditions can sustain large negative pressures for a prolonged time before cavitating. In other words, stretched water is trapped in a metastable state and bubble nucleation takes place as a rare event. In fact, in the appropriate thermodynamic conditions, the cavitation nucleus spontaneously appears due to thermal fluctuations. However, the probability that a fluctuation able to trigger the transition takes place could be extremely rare. This implies that the time needed to observe cavitation can be quite long not only on the atomistic scale. For instance, at the temperature of $50\,^\circ \mathrm{C}$ and pressure $p = -70 \, \mathrm{MPa}$, the nucleation rate (number of nucleated bubbles per unit time and volume) is $J \simeq 10^{-36}\, \mathrm{s}^{-1} \mathrm{m}^{-3}$. This figure can be translated into an average time of 30 billion years (to be compared with the age of the Universe estimated in 13.77 billion years) to wait before observing a single bubble in a volume corresponding to the water content of the oceans (assuming they cover 70% of the Earth surface with a mean depth of $3700\, \mathrm{m}$ and were formed by pure water). Slightly oversimplifying, the liquid-vapor transition implies two states, one of which—the liquid—is metastable and the other one—the vapor—which is the thermodynamically stable state, corresponding to the absolute free energy minimum. The two states are separated by a free-energy barrier with a critical state across which the system passes when transitioning. In fact the free-energy barrier and, as a consequence, the nucleation rate strongly depend on temperature and pressure, easily spanning a range of one hundred orders of magnitude, with the transition becoming quite fast at spinodal conditions.

In the last decades, different experimental techniques were conceived to determine the water stability limit, such as aqueous inclusions in quartz^1,2^ and acoustic experiments^3,4^. It is now established that the typical pressure to observe vapor bubble formation in ultra pure water at ambient conditions within a reasonable time window is on the order of −120 MPa^5^. The cavitation pressure $p_{cav}$ increases with temperature up to $\simeq 22 \, \mathrm{MPa}$ in critical conditions ($T_{c} = 373.94 \, ^\circ \mathrm{C}$). At high temperatures ($T \simeq 300 ^\circ \mathrm{C}$) there is broad consensus among the different experimental techniques on the estimated cavitation pressure^6^. However, discrepancies are observed in the range of low temperatures. The issue is relevant for the long lasting debate on the anomalies of water, namely the shape of the line of maximum density which, according to different scenarios, may or may not intersect the spinodal line^5,7–9^.

Classical Nucleation Theory (CNT) provides the basic framework to understand the nucleation process. It assumes a uniform state inside the spherical bubble up to the dividing surface which separates the vapor from the external uniform liquid. The (free-)energy of the interfacial layer is ascribed to the zero thickness interface leading to a simple free-energy model consisting of bulk part and interfacial contribution. Near saturation conditions, where the bubble radius is sufficiently larger than the thickness of the interfacial layer, CNT provides qualitatively correct results. However, the CNT nucleation barrier is found to be overestimated leading to large discrepancies with the experimentally observed cavitation rates and cavitation pressures^6,10^.

Recently, Menzl et al.^11^ corrected the CNT by including the Tolman length $\delta$ in the basic model to account for the effect of interface curvature on the surface tension $\sigma$. Assuming $\delta > 0$, as generally accepted, the curvature decreases $\sigma$ and the nucleation free-energy barrier, $\Delta \Omega ^* = 16/3 \pi \sigma _*^3/\Delta p^2$, is decreased. Since the barrier height controls the escape rate from the potential well, an increase of the nucleation rate by orders of magnitude follows.

In^11^, the classical Kramers approach was complemented by determining the diffusion coefficient in the Langevin equation for the bubble volume on the basis of the overdamped Rayleigh–Plesset equation for the nucleating bubble. This extended approach, completed with data from molecular dynamics (MD) simulations proved successful in reproducing the order of magnitude of the cavitation pressure estimated from quartz inclusion experiments at ambient temperature^5^.

In the present paper we attack the problem of homogeneous vapor bubble nucleation in water with a Diffuse Interface (DI) approach^12^ that we recently developed for model fluids (Van der Walls equation of state or Lennard–Jones fluids)^13–17^. Historically, the DI model originated from the pioneering work on capillarity of Van der Waals who introduced a continuous, sharply varying density distribution $\rho (\mathbf{r})$ in place of a sharp interface. This approach is a simplified version of density functional theory (DFT). The purpose of the proposed model is to reproduce the two crucial observables related to water cavitation, namely the nucleation rate *J*, defined as the number of bubbles nucleated per unit time and volume, and the cavitation pressure $p_{cav}$. Given the volume of the liquid sample, $V_l$, the latter is defined as the liquid pressure for which the probability to observe at least one cavitation bubble in a given time window, $0 \ge t \ge \tau$, equals 1/2. This definition is based on assuming nucleation as a random Poisson point process^18^, where single nucleation events are independently distributed in space and time.

The DI model is completed with a realistic equation of state for water—the IAPWS-95 EoS^19^—and the surface energy of the planar interface, $\sigma _0(T)$, as a function of temperature^20^. The model implicitly includes the effect of interface curvature on the surface energy, accounting for the Tolman length, $\delta$ (see “Methods”).

## Results

**Figure 1:**
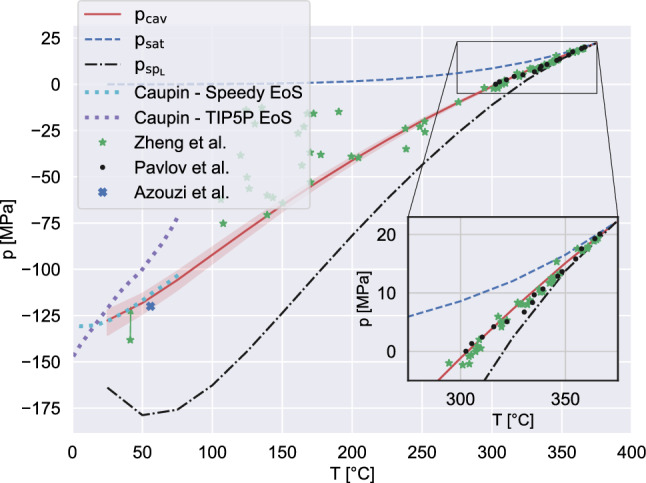
Cavitation pressure as a function of temperature. The red solid line reports the values as predicted by the diffuse interface model, as explained in the text; the light red band shows the sensitivity to the experimental parameter $V_l \tau = 1000 \, \upmu \mathrm{m}^3 \cdot \mathrm{s}$ when changed by a factor $10^{-3}$ (lower limit) and $10^3$ (upper limit). The dash-dotted black, and the dashed blue, lines show the spinodal and saturation pressures, respectively, as provided by IAPWS EoS. The different symbols (green stars^2^, black dots^21^) represent available experimental data. The arrow indicates the correction due to the matrix compliance effect. The two dotted curves report data from^22^ exploiting two different water EoS, as indicated in the legend. The inset zooms into the high temperature region, close to the critical point.

One of the main results of the paper is provided in Fig. 1 which shows the cavitation pressure of ultra-pure water as a function of temperature. Introducing the nucleation rate *J*, the average number of nucleated bubbles observed in a liquid volume $V_l$ within the time window $0 {--} \tau$ is, by definition, $\langle n \rangle = J V_l\tau$. Given the Poisson distribution of the bubble number $\pi (n | V_l, \tau ) = 1/n! (J V_l \tau )^n \exp (-J V_l \tau )$, the probability of having at least one bubble is $1-\exp (-J V_l \tau )$. Based on the above assumptions, the cavitation pressure is the liquid pressure at which this probability is exactly 1/2, i.e. $J(p_{cav}) = \ln (2)/(V_l \tau )$.

The data shown by the red thick curve in the figure were obtained by evaluating, for given temperature, the nucleation rate—see “Methods”—for different pressures to extract $p_{cav}$ by interpolation. Within the shaded region, obtained by varying $V_l \tau$ in the range $1 \div 10^6 \, \upmu \mathrm{m}^3 \cdot \mathrm{s}$, the cavitation pressures changes by less than $10\%$ for all the analyzed temperatures, confirming the robustness of this observable.

The predictions of the present model compare favorably with the experimental data at high temperature^2^ (quartz inclusions)^21^, (heat pulse method), see also the figure inset. As the temperature is reduced, the experimental scatter on the cavitation pressure data increases considerably. The point indicated by the blue symbol on the left of the plot concern a single, very accurate measurement taken with quartz inclusions^5^. Azouzi et. al., after measuring the water density $\rho _L = 922.8 \, \mathrm{kg/m}^3$ and the temperature $T \simeq 55^\circ \mathrm{C}$, obtained the cavitation pressure $p_{cav} \simeq -120\, \mathrm{MPa}$. To evaluate the pressure, they used the same IAPWS-95 EoS of our present numerical simulations which yield $p_{cav} \simeq -118\, \mathrm{MPa}$ at $T \simeq 50^\circ \mathrm{C}$.

**Figure 2:**
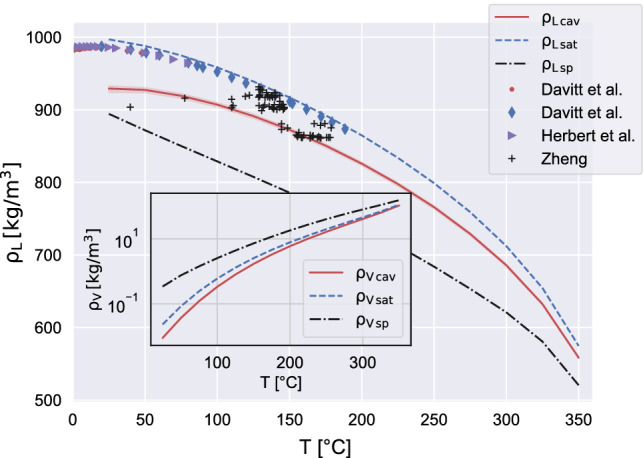
Liquid density at cavitation as a function of temperature. The curve colors and styles are the same as in Fig. 1. The available experimental data are obtained with different techniques: red circles^4^, blue diamonds^4^ and purple triangles^3^ are obtained with acoustic experiments; the black plus signs^23^ are from inclusion experiments. More specifically, red circles are directly measured from fibre optic probe hydrophone, while purple triangles and blue diamonds by converting into density the $p_{cav}$ estimated with the static pressure method. The inset reports the corresponding density at the vapor bubble centre with the solid red curve, compared with spinodal (dash-dotted black) and saturation (dashed blue).

Figure 2 provides the liquid mass density at cavitation as a function of temperature, namely the density of the liquid far away from the bubble (i.e. in the bulk liquid state corresponding to the assigned temperature and pressure). The numerical data shown by the red line correspond to those given in terms of pressure in the previous Fig. 1. The experimental data come from four different experiments. The plus symbols^23^ provide cavitation density data from quartz inclusion experiments. They substantially correspond to the pressure data denoted by green stars^2^ in the pressure diagram. The left-most point is presumably affected by some inaccuracy^2^ and it is expected to underestimate the pressure, see the vertical green arrow in Fig. 1. The remaining experimental data points, see caption, provide data from acoustic experiments, which are believed to anticipate the cavitation. The inset shows the vapor density at the bubble center, an information which is unaccessible to experiments. Our model shows that vapor is close to saturation conditions, confirming expectations and CNT assumptions.

**Figure 3:**
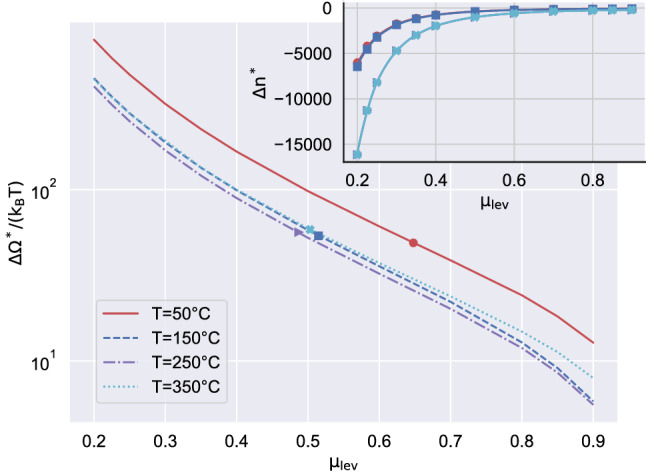
Free energy barrier $\Delta \Omega ^*$, normalized with the thermal energy $k_B T$, as a function of the metastability level $\mu _{lev} = (\mu - \mu _{sat})/(\mu _{sp} - \mu _{sat})$ at four different temperatures. The symbols represents the values where $p=p_{cav}$ at those temperatures. The (negative) excess number of molecules in the critical bubble, $\Delta n^*$, as a function of the metastability level, is shown in the inset at the same temperature of the main plot, with corresponding colors. The symbols correspond to the data obtained with the diffuse interface model by integrating the density profile of the critical bubble, $\Delta n^* = (1/m)\int _0^\infty (\rho _c(r)- \rho _L)r^2\,{\mathrm d}r$, with *m* the mass of a water molecule. The solid lines are evaluated by applying the Nucleation Theorem^30^ as $\Delta n^*=-(\partial \Delta \Omega ^*/\partial \Delta \mu )/m$. Please notice that purple (red) curves and symbols are almost totally hidden by the light blue (rep. dark blue) ones.

As explained in “Methods”, the string method is used to find the critical state. Briefly, the string method^24,25^ is a specialized approach to extract the minimum free energy path in the transition between (meta)stable states and allows to determine the free-energy barrier. This class of algorithms is suggested by the rare event nature of the transition process which, under certain circumstances, can be extremely slow, hindering the adoption of direct simulation strategies. The nucleation free-energy barriers for different temperatures in the range 50–350$^\circ \,\mathrm{C}$ are plotted in Fig. 3 as a function of the metastability level, $\mu _{lev} = (\mu - \mu _{sat})/(\mu _{sp} - \mu _{sat})$^26^, with $\mu$ the chemical potential and the subscripts *sp* and *sat* denoting spinodal and saturation conditions, respectively. The $\Delta \Omega ^*$ vs $\mu _{lev}$ curves tend to collapse, although not exactly, at high temperature, a feature consistent with the density functional theory (DFT) results reported in^26^ for a Lennard–Jones fluid. In the present case, this behavior is apparently violated when the temperature decreases, red curve. The symbols in the main plot identify the barrier at the cavitation pressure, i.e. $\mu _{lev} = (\mu (p_{cav}, T) - \mu _{sat}(T))/(\mu _{sp}(T) - \mu _{sat}(T))$. Clearly, the barrier in thermal units at cavitation conditions is substantially independent of temperature, owing to the definition of cavitation pressure ($J(p_{cav}) = \ln (2)/(V_l \tau )$) and the expression of the nucleation rate $J = \Gamma _0 \exp (-\Delta \Omega ^*/(k_B T))$, which implies that the temperature dependence of $\Delta \Omega ^*$ is given by the additive contribution $\ln (\Gamma _0)$, where $\Gamma _0(T)$ is, e.g., the Blander and Katz^27^ prefactor.

In a famous paper, Kashchiev^28^ derived the celebrated nucleation theorem, $(1/m) \partial \Delta \Omega ^*/\partial \Delta \mu = - n^*$, where $n^*$ is the molecule number in the critical cluster, with *m* the molecule mass, stating that it is expected to hold for classical, atomistic, homo- or heterogeneous, three- or two-dimensional nucleation. A more general result has been obtained in^29^ where the theorem has been proved in the context of DFT, with continuously varying density fields. In^26^ the original expression was modified by substituting the molecule number with the excess/defect of molecules, $\Delta n^*$, to extend its range of application to bubbles.

The nucleation theorem is shown to be preserved also in the present DI context, as shown in the inset of Fig. 3 which plots the defect molecule number as a function of the metastability level. The symbols are a direct measure of the defect number (RHS of the nucleation theorem), while the curves provide the derivative of the barrier (LHS). Data are reported for the four temperatures of the main plot, with the curves hiding each other in couples.

The DI approach gives access to the surface tension, $\sigma (T)$, see “Methods”, Eq. (8). The theory naturally includes the effect of interface curvature, providing $\sigma (T)$ as a function of the bubble radius, defined in this context as

1 $$\begin{aligned} R= \int _0^\infty r \left( \dfrac{d\rho }{dr}\right) ^2 dr/\int _0^\infty \left( \dfrac{d\rho }{dr}\right) ^2 dr. \end{aligned}$$

**Figure 4:**
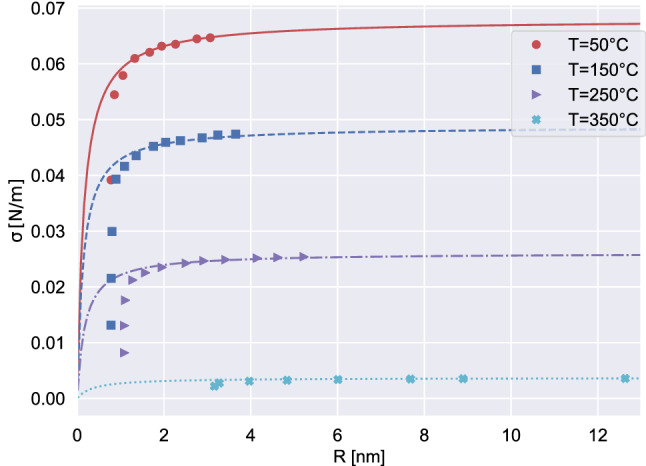
Liquid-vapor surface tension as a function of bubble radius *R* at four different temperatures. The data obtained via DI model are shown with symbols. The lines report the fits with the Tolman equation $\sigma (R, T) = \sigma _0(T)/(1 + 2\delta (T)/R)$ that minimises the differences between the nucleation rates obtained with the DI model and with the corrected-CNT^11^, as explained in the dedicated subsection of “Methods”. The optimal $\delta$ values are $\{0.07582, 0.06835, 0.08888, 0.18412\} \mathrm {nm}$ at temperatures $\{50, 150, 250, 350\} ^\circ \mathrm {C}$, respectively.

For each temperature, the data shown in the Fig. 4, symbols, are taken from the critical bubble at different metastability level. The lines are the fitting of the surface tension using the Tolman expression, $\sigma (T,R) = \sigma _0/(1 + 2 \delta /R)$, where the parameter $\delta$ is the Tolman length^31^ and $\sigma _0$ is the surface tension of the planar interface. We stress that $\sigma _0$ at the different temperatures are taken from the fitted IAPWS expression for water^20^. This uniquely determines the capillary parameter entering the DI free energy, $\lambda (T)$, *Methods*. Apparently, the behavior of the surface tension as a function of the critical bubble radius is well described by the Tolman fit, at least for reasonably large bubbles.

**Figure 5:**
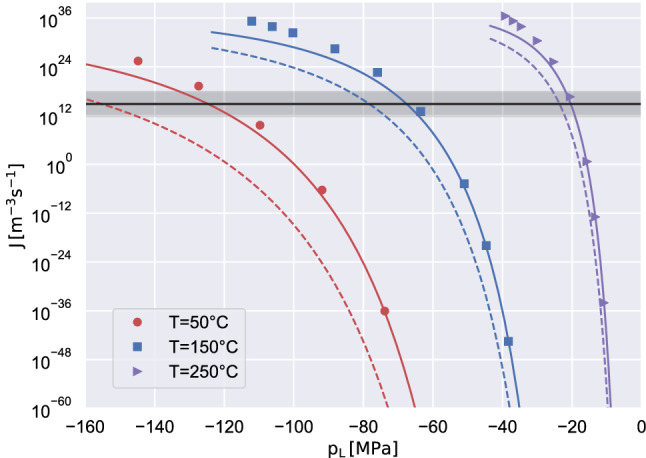
Bubble nucleation rate *J* as a function of the metastable liquid pressure for different temperatures. The DI data are reported with symbols, while the dashed and solid curves correspond to the predictions of the plain-CNT and the Tolman-corrected-CNT, respectively. The horizontal black line indicates the nucleation target rate $J_{1/2} = \text {ln}2/(V_l\tau )$, corresponding to the value of the rate such that one bubble is observed in a system of volume $V_l$ over an observation time $\tau$ with probability 1/2, following Ref.^6^. The black light band shows the sensitivity to the experimental parameters $V_l \tau = 1000 \upmu \mathrm{m}^3 \times 1s$ by a factor of 0.001 (upper band) and 1000 (lower band).

Beside the cavitation pressure, the nucleation rate *J* is the other most important observable. The classical Kramers’ theory^32^ provides the reaction rate by modeling the process as the escape of a random walker from the free-energy potential well. Figure 5 shows *J* as obtained from the barrier heights taken from the present DI model using the prefactor $\Gamma _0 = (\rho _L \rho _V/m^2) \sqrt{k_B T \sigma ^3}/(\eta \Delta p)$. The solid lines in Fig. 5 show, for comparison, the data obtained using the Tolman-corrected CNT^11^ while the broken lines correspond to the plain CNT.

## Discussion

The diffuse interface method adopted in the present paper assumes the free energy of the capillary system as described by a functional of the density field $\rho (\mathbf{r})$ expressed as a volume integral of the bulk free-energy density and a capillary contribution proportional to the squared density gradient, see “Methods”. The equilibrium density field presents a strong gradient, sharply raising from the vapor to the liquid density at the interface between the two phases. The model can be interpreted as a gradient expansion of the functional used in DFT^33^. In can be considered as an effective theory where a few basic ingredients need to be included, namely the equilibrium thermodynamic properties of the liquid/vapor system. This allows the flexibility required to model realistic fluids, in the present case water, as described by the IAPWS EoS^19^ for bulk free energy and surface tension, in a wide range of temperatures and pressures. In the model the EoS should encompass all the states along the transition between the two phases (from the metastable liquid to the stable vapor in the cavitation bubble). In particular, also the unstable states below the spinodal line need to be considered. This raises an issue, since no direct experimental information is available in the unstable region. As discussed in the “Methods”, the procedure we have adopted consists in extrapolating data from the EoS to spinodal conditions and connect the limiting states across the unstable region. The interpolation is achieved with the constraints that (i) the pressure should be a strictly decreasing function of density at constant temperature (to prevent unphysical regions of local stability below the spinodal line), (ii) $\partial p/\partial \rho (\rho _{sp}) \vert _T = 0$ (as required for spinodal states), and (iii) enforcing the continuity of pressure and free-energy. This procedure leaves a large freedom on the choice of the specific interpolation function. As shown in the Supplementary Information (SI), the model is insensitive to the details in the unstable region. The sensitiveness is larger to modifications of the shape of the spinodal curves, SI Fig. 2. In^22^, using an approach similar to the present one, the effect on cavitation of the topology of the spinodal line was discussed in some detail, concluding that a so-called reentrant spinodal, as in the case of the IAPWS EoS, is presumably better suited to capture cavitation data.

As shown in the “Results” section, our simulations reproduce remarkably well the best data available for bulk water. In comparing simulation data with experiments, one should be aware of certain technical difficulties encountered on the experimental side. As the authors of the experiments themselves underline, in certain cases, particularly at low temperature, it turned out difficult to strictly control the purity of the water sample. This is especially true of experiments done with acoustic excitations^3,4^. Water inclusions in quartz^1,2^, on the other hand, reduce the probability that impurities are present in the sample by using an extremely small volume of water, exploiting the quartz hydrophilicity to prevent bubble nucleation at the solid interface, which would lower the transition barrier. Nevertheless, each particular sample showed a different behavior in terms of cavitation pressure. Substantial effort was put in identifying the most suitable sample to perform repeated nucleation experiments in order to extract statistically reliable data^5^. Based on these considerations, the experimental data should, in general, be interpreted as overestimating the actual cavitation pressure pointing the attention to the low pressure envelope of the available data. With these comments in mind, the present simulation data are entirely consistent with experiments throughout the extended range of temperatures from 25 to 350$^\circ \, \mathrm{C}$ where reliable experimental data are available.

As discussed in the introductory material, Kramers’ theory was revised and adapted to bubble nucleation in a recent paper^11^ where the Tolman length and the flat interface surface tension were fitted on MD data obtained with the TIP4P water model, predicting the cavitation pressure of $-126 \, \mathrm{MPa}$ at the temperature $T = 23.25^\circ \,\mathrm{C}$. We like to stress that the Tolman length $\delta$ is implicitly included in the present DI model, where the density distribution $\rho ({\mathbf{r}})$ is influenced by the size, hence the curvature, of the vapor bubble. As a consequence the excess grand potential that controls the actual surface tension felt by the system depends on bubble curvature. Figure 4 shows that the surface tension of the critical nucleus can be fitted remarkably well by the Tolman expression (see the caption for the values of $\delta$), at least in conditions where the interfacial thickness remains sufficiently small with respect to the bubble size for the very concept of Tolman length to make sense. As a consequence, at the temperature of $25^\circ \,\mathrm{C}$ our model predicts $p_{cav} = -127.6 \, \mathrm{MPa}$ without introducing additional information apart from the EoSs, Fig. 1.

The thickness $\ell$ of the interfacial layer is amenable to direct measurement with X-ray scattering and ellipsometry^34,35^ respectively. The related data obtained from the present model are listed in the SI, Table 2, where the possibility to alter the interfacial thickness for given surface tension by changing the EoS in the unstable region is also briefly addressed. In fact, the interfacial thickness may easily be changed by $15 \%$ with no alteration of the nucleation rate and cavitation pressure.

As a final remark, we stress that several different prefactors, see, e.g.^11,27^, may be considered in the expression of the cavitation rate. The cavitation pressure is confirmed to be almost independent of the specific prefactor (SI, Fig. 3).

## Conclusions and perspectives

Modeling cavitation in water has longly been an elusive issue. In this paper we have shown in detail how a diffuse interface method completed with realistic equations of state for bulk water and for the vapor-liquid surface tension can be exploited in combination with rare event techniques to evaluate the free-energy barrier.

The model is shown able to capture the experimental values of the cavitation pressure in water over the wider range of temperatures over which experimental data are available, e.g. from $25^\circ \mathrm{C}$ to critical conditions. Our data provide a lower envelope for the low temperature data, which are known to overestimate the cavitation pressure and the particularly accurate low temperature (ambient) data by^5^ are well matched by the present predictions.

We also confirm the findings discussed in^11^ where the authors show that the effect of interfacial curvature is crucial to reproduce realistic nucleation barriers. We stress, however, that the Tolman parameter is not a fitting degree of freedom in the present model, which is completely determined by equation of state and planar interface free-energy. The present data allow to directly verify the validity of the Tolman correction which, as shown in Fig. (4), works perfectly well for larger sizes, deteriorating progressively until becoming unsuitable as the size of the critical nucleus approaches the interfacial layer thickness. In principle, the data can be used to find more general fitting procedures for the surface tension as a function of bubble curvature see, e.g.^36^ for a more complete discussion based on experimental data.

The DI model is highly flexible and, in principle, can be extended to include the effect of dissolved gases in the metastable liquid or heterogenous nucleation over different kinds of surfaces^14^. An important feature of the DI model that was up to now exploited only for model fluids (Van der Waals EoS and Lennard–Jones fluids) is the possibility to describe the dynamic evolution of the cavitation bubble coupled with the inertial effect associated with liquid motion, to the point that even extreme events like shock waves launched in the liquid at bubble collapse, both on free space and near boundaries, can be addressed^37–39^. The dynamic approach can be extended in the spirit of Landau and Lifshitz’s fluctuating hydrodynamics to include the effect of thermal fluctuations^13,14^ and may also account for the wettability of solid boundaries and the coupling with macroscopic flow motion. Finally, the resulting system of stochastic partial differential equations allows to deal with systematic heat injection for heat transfer problem.

## Methods

### Diffuse interface model

The liquid-vapor system is described via a Diffuse Interface (DI) modelling based on the square gradient approximation (SGA) of the (Helmholtz) free energy functional, proposed in a seminal work by van der Waals^40^. This approach is understood as a particular case of the more general Density Functional Theory (DFT)^33^ and allows to control the thermodynamic behavior of the fluid through the choice of effective equations of state expressed in terms of the bulk free energy density, $f_b(\rho , T)$. The total (Helmholtz) free energy functional is

2 $$\begin{aligned} F[\rho , T] = \int _{{\mathcal {V}}} f_b (\rho , T) + \dfrac{\lambda (T)}{2} \vert \varvec{\nabla } \rho \vert ^2 \, \mathrm {d}V, \end{aligned}$$

where $\lambda (T)$ is the temperature dependent capillary coefficient that enables to tune the surface tension of the flat liquid–vapor interface $\sigma _0(T)$ of the specific fluid at any given temperature, the surface tension of water^20^ in the present work. More precisely, $\sigma _0(T)$ is evaluated as:

3 $$\begin{aligned} \sigma _0(T) = \int _{-\infty }^{+\infty } \lambda \left( \dfrac{\partial \rho _{eq}}{\partial x}\right) ^2 \,{\mathrm d}x = \int _{\rho _V^{sat}(T)}^{\rho _L^{sat}(T)} \sqrt{2 \lambda \left[ \omega _b(\rho , T) - \omega _b(\rho _V^{sat}(T),T) \right] } \,\mathrm {d} \rho , \end{aligned}$$

expressed both in terms of the equilibrium density profile $\rho _{eq}(x)$ across the flat interface, or directly through the EoS (see^39^ for details). In the above equations, $\omega _b(\rho , T) = f_b(\rho , T) - \mu _b(\rho , T) \rho$ is the grand potential density, $\mu _b(\rho , T) = \partial f_b/\partial \rho$ is the chemical potential and $\rho _{V/L}^{sat}(T)$ are the temperature dependent vapor and liquid densities at saturation, respectively. The specific procedure to adapt the IAPWS-95 EoS (the actual expression of free energy $f_b(\rho , T)$ used to reproduce the water thermodynamic properties) to the DI approach is described in the next subsection.

### Free energy barrier evaluation

As discussed in the main text, nucleation is a rare event which calls for specialized algorithm to determine the free energy barrier and, more generally, the minimum free energy path. The total free energy functional is minimized, with the constraint of given total mass, to obtain the equilibrium condition

4 $$\begin{aligned} \mu _b(\rho , T) - \lambda (T) \nabla ^2\rho = \mu _{ext}, \end{aligned}$$

where the external chemical potential, $\mu _{ext}$, and the system temperature are assigned. The above condition implies that the generalized chemical potential, including the capillary term, is spatially homogeneous at equilibrium. When the external chemical potential corresponds to a metastable liquid condition, one possible (unstable) equilibrium solution is the critical bubble immersed in the metastable liquid, described in terms of the critical density profile, $\rho _c(r)$ with *r* the radial coordinate originating at the bubble center. In this work, the powerful string method^24,25^ is deployed to obtain the density profile of the critical bubble, corresponding to the saddle point of the (Landau) free energy landscape, i.e. the transition state^13,16^. In a nutshell, the string method numerically approximates the minimum energy path (MEP) describing the continuous sequence of density configurations, $\rho (r, \alpha )$, along the transition path parametrized by the transition coordinate $\alpha$. The images $\rho ^k(r)$ forming the string are evolved over the pseudo-time ${\tilde{t}}$ according to the steepest-descent algorithm

5 $$\begin{aligned} \dfrac{\partial \rho ^k}{\partial {{\tilde{t}}}} = \mu _{ext} - \left[ \mu _b(\rho ^k) - \dfrac{\lambda }{r^2}\dfrac{\partial }{\partial r}\left( r^2\dfrac{\partial \rho ^k}{\partial r}\right) \right] , \end{aligned}$$

representing a relaxation evolution of Eq. (4), written in spherical coordinates. A reparametrization procedure redistributes the images after evolving Eq. (5) over a single pseudo-time step $\Delta {{{\tilde{t}}}}$. This two-steps procedure is iterated up to the complete convergence of the whole string to the MEP. The configuration $\rho _c(r)$ laying on the saddle point corresponds to the critical bubble. The relaxation dynamics of the string images, Eq. (5), is implemented by a centered, second order accurate finite difference scheme, with (pseudo-)time advancement performed by a forward Euler scheme. The forward scheme is used for its simplicity and stability properties, considering the time accuracy is not an issue here since we only need the steady, fully relaxed solution.

The critical radius, the energy barrier and the curvature dependent surface tension, are then measured following^41^ as

6 $$\begin{aligned}&R^*= \int _0^\infty r \left( \dfrac{d\rho _c}{dr}\right) ^2 {\mathrm d}r\Big /\int _0^\infty \left( \dfrac{d\rho _c}{dr}\right) ^2 {\mathrm d}r, \end{aligned}$$

7 $$\begin{aligned}&\Delta \Omega ^*= 4\pi \int _0^\infty \left\{ f_b(\rho _c(r))-f_b(\rho _L) -\left[ \rho _c(r)-\rho _L\right] \mu _{ext} + \dfrac{\lambda }{2} \left( \dfrac{d\rho _c}{dr}\right) ^2 \right\} r^2{\mathrm d}r, \end{aligned}$$

8 $$\begin{aligned}&\sigma ^*= \left[ \dfrac{\lambda }{4}\int _0^\infty \left( \dfrac{d\rho _c}{dr}\right) ^2 r^2{\mathrm d}r\right] ^{1/3} \left[ f_b(\rho _L)-f_b(\rho _c(0)) -(\rho _L-\rho _c(0))\mu _{ext}\right] ^{2/3}, \end{aligned}$$

respectively (the temperature *T*, being a parameter, has been avoided to ease the notation).

### Equation of state

The IAPWS-95 EoS^19^ is here used to accurately describe the thermodynamic properties of water, both in the liquid and the vapor phases. The EoS provides an empirical expression for $f_b(\rho ,T)$ obtained by fitting a large experimental dataset of stable liquid and vapor states. The values at metastable conditions are extrapolated up to the spinodal states, where the condition $\partial p/\partial \rho = 0$ is met. In the whole unstable region, $\rho _{sp\, V}<\rho <\rho _{sp L}$, the EoS exhibits spurious oscillations, preventing the direct application into the DI model. In fact, the presence of density intervals where $\partial p/\partial \rho > 0$ in the unstable region, produces unphysical stable-states. The original $f_b(\rho ,T)$ is corrected by modifying the expression in the unstable region at each temperature:

9 $$\begin{aligned} f_b^{corr}(\rho ,T) = \left\{ \begin{array}{ll} f_b(\rho ,T), \quad &{} \rho \le \rho _{sp V}(T) \cup \rho \ge \rho _{sp L}(T) \\ f_b^{mod}(\rho ,T),\quad &{} \rho _{sp\, V}(T)< \rho < \rho _{sp L}(T) \end{array}\right. . \end{aligned}$$

$f_b^{mod}$ is obtained by enforcing the following six conditions requiring the continuity of the pressure and its derivative and of the free energy at the spinodal states:

10 $$\begin{aligned} \left\{ \begin{array}{l} p^{mod}(\rho _{sp V}) = p(\rho _{sp V}) \\ p^{mod}(\rho _{sp L}) = p(\rho _{sp L}) \\ \dfrac{\partial p^{mod}}{\partial \rho }(\rho _{sp V}) = 0 \\ \dfrac{\partial p^{mod}}{\partial \rho }(\rho _{sp L}) = 0 \\ f_b^{mod}(\rho _{sp V}) = f_b(\rho _{sp V}) \\ f_b^{mod}(\rho _{sp L}) = f_b(\rho _{sp L}) \end{array}\right. . \end{aligned}$$

The following third order polynomial supplemented with an exponential function is used as a prototype function for the modified pressure, $p^{mod} = A \rho ^3 + B \rho ^2 + C\rho +D + E \exp (\rho )\rho ^2$. The cubic expression guarantees that no spurious oscillations occur in the unstable region. The sixth free parameter, *F*, appears as the integration constant when integrating $p^{mod}$ to obtain the free energy, as $f_b^{mod} = \int (p^{mod}/\rho ^2) {\mathrm d}\rho + F$. Other possible choices for $f_b^{mod}$ have been tested, showing a very weak sensitiveness on the obtained cavitation pressure (Supplementary Information (SI)).

### Tolman fit

An iterative best fitting procedure has been exploited to estimate the Tolman length from the DI surface tension $\sigma ^*$ in critical conditions, Eq. (8). For each temperature *T*, the data consist of the set $\{\sigma ^*_i = \sigma ^*(\mu _{lev}^i, T)\}$, measured at the *i*-th metastability level ($i=1,M$). They are plotted, for several temperatures, as a function of the corresponding critical radius, $R^*_i$, in Fig. 4. Iterating over *n*, at fixed *T*, the Tolman length $\delta _n$ is obtained by using the Tolman law as fitting function $\sigma ^*_i(T) = \sigma _0(T)/(1 + 2\delta _n(T)/R^*_i)$, with $\sigma _0(T)$ the temperature dependent water surface tension of the flat interface provided by^20^. At the *n*-th iteration level, the Tolman length $\delta _n$ is obtained by employing the subset $\{\sigma ^*_i(T)\}$ with *i* from $n+1$ to *M* thus progressively eliminating from the fit the data at the smallest radii. The “corrected”-CNT, with the curvature dependent surface tension, has then been applied to estimate the nucleation rates $J_n(i) = J_n(\mu _{lev}^i, T)$ at the different metastable liquid conditions, by employing the different $\delta _n$ fitting values. The optimal Tolman length, $\delta _{opt}(T)$, is the one that minimizes the L2 norm of the difference between the “corrected”-CNT estimate and the rate measured from the DI model, $|| \log _{10} J_n - \log _{10} J^{DI} || = (\sum _{i=1}^M |\log _{10} J_n(i) - \log _{10} J^{DI} (i)|^2)^{1/2}$. The fitting error for the different *n* and several temperatures are reported in the SI. The caption of Fig. 4 reports the optimal Tolman lengths at the different temperatures considered in the plot.

### Nucleation rate

The evaluation of the nucleation rate in the different thermodynamic conditions follows the line of the Kramers’ theory^32^, providing the transition rate of the nucleation process as the escape of a random walker from the free-energy potential well, $k = [(\int _\cup \exp (-\beta \Delta \Omega (x))dx)(\int _\cap \exp (\beta \Delta \Omega (x))/D(x)dx)]^{-1}$, along the transition coordinate *x*. In this expression $\beta =1/(k_B T)$, *D*(*x*) is the diffusion coefficient, and $\cup ,\cap$ are shortcuts to indicate that the integration is performed over the the well and barrier basin, respectively. The corresponding nucleation rate is then evaluated as $J = \Gamma _0 \exp (-\Delta \Omega ^*/(k_B T))$, with the prefactor $\Gamma _0 = c_0 \sqrt{k_B T \sigma ^3}/(\eta \Delta p)$ derived in^11^ in the case of bubble nucleation. The normalization constant $c_0$ is not uniquely defined in the framework of CNT, but we adopted the expression $c_0=\rho _L\rho _V/m^2$^27^ which compared favorably with the value obtained with MD in^11^. It is worth stressing that the specific expression, formally resembling that of plain-CNT, contains the distinctive feature that the energy barrier, the surface tension and the pressure jump, are all evaluated from the critical density profile $\rho _c(r)$ obtained with the string method (Eqs. 6–8). When comparing the rates in Fig. 5, the plain-CNT prediction is obtained by substituting in the above expression the surface tension of the flat interface $\sigma _0$, $\Delta \Omega ^* = 16\pi \sigma _0^3/(3 \Delta p^2)$, and $\Delta p = p_{sat}-p_L$. Conversely, in the Tolman-corrected-CNT, the curvature dependence of the surface tension $\sigma (R)=\sigma _0/(1 + 2\delta /R)$ is considered in the derivation of the specific expressions^11^, while the value of the Tolman length $\delta$ is extracted from our data, as explained in the previous subsection.

## Supplementary Information

Supplementary Information.
