# Supplementary material for: Water cavitation from ambient to high temperatures
Source: Sci Rep. 2021 Oct 21;11:20801. doi: 10.1038/s41598-021-99863-z (PMC8531334; doi:10.1038/s41598-021-99863-z)
Supplement: Supplementary file 1 — Supplementary Information. [file 41598_2021_99863_MOESM1_ESM.pdf]

Supporting Information "Water cavitation from ambient to high temperatures"

Francesco Magaletti, Mirko Gallo, and Carlo Massimo Casciola

1 Equation of State for water

In this work the IAPWS-95 EoS [1] is used to reproduce the properties of water in both phases. The EoS is expressed in terms of the free-energy density $f_b(\rho, T)$ obtained by fitting a large experimental dataset of stable liquid and vapor states. The values at metastable conditions are extrapolated to the spinodal states, where the condition $\partial p/\partial\rho = 0$ is met. In the whole unstable region, $\rho_{sp\,V} < \rho < \rho_{spL}$, the original EoS exhibits spurious oscillations producing unphysical stable-states. This prevents the direct application of the EoS into the DI model. The original $f_b(\rho, T)$ is corrected by modifying the expression in the unstable region at each temperature. The main requirement is $\partial p/\partial\rho < 0$ in the whole unstable region. A different possibility, with respect to the easier choice adopted in the main text, is the family of functions such that

$$\frac{\partial p^{mod}}{\partial \rho} = -A(\rho - \rho_{spV})(\rho_{spL} - \rho)\exp[-n(\rho - B)^2]\,, \tag{1}$$

$$p^{mod}(\rho) = \int_{\rho_{spV}}^{\rho} \frac{\partial p^{mod}}{\partial \rho'}\mathrm{d}\rho' + p(\rho_{spV})\,, \tag{2}$$

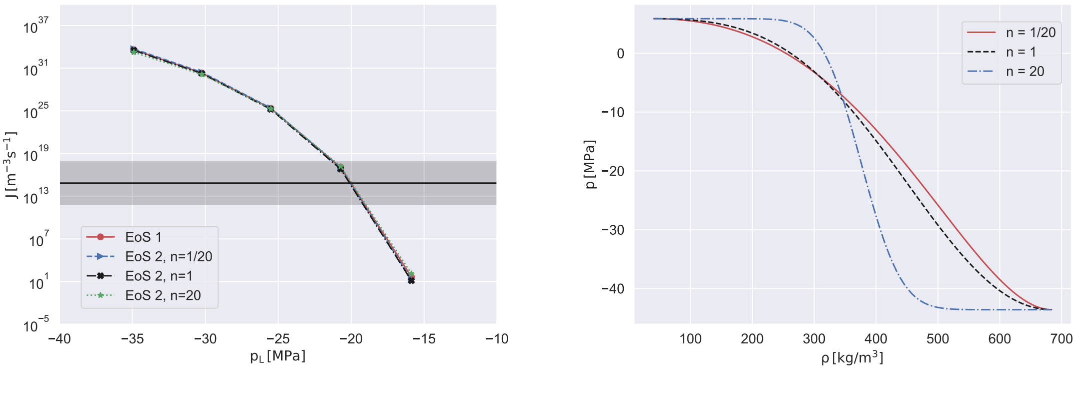

Figure 1: Left panel: Comparison of the nucleation rate obtained at $T = 250°\mathrm{C}$ employing the modified EoS adopted in the main text (EoS1) and the family of functions defined in Eqs. 1–3 (EoS2). The free parameter n is varied by few order of magnitudes. The results show an almost complete insensitivity on the nucleation rate, notwithstanding the strong difference in the density dependence of the pressure (right panel).

n	$\ell_{10-90}(T=250°\mathrm{C})$[nm]	$\ell_{10-90}(T=50°\mathrm{C})$[nm]
1/20	1.598	0.987
1	1.545	0.914
20	1.404	0.798

Table 1: Interface thickness ℓ_{10-90} at two different temperatures.

and

$$f_b^{mod}(\rho) = \int_{\rho_{spV}}^{\rho} \frac{p^{mod}(\rho')}{\rho'^2}\mathrm{d}\rho' + f_b(\rho_{spV})\,. \tag{3}$$

In the first definition, Eq. 1, n is a given positive constant and A, B are two parameters to be identified enforcing the values of the spinodal liquid pressure, $p^{mod}(\rho_{spL}) = p(\rho_{spL})$, and the corresponding Helmholtz free energy density, $f_b^{mod}(\rho_{spL}) = f_b(\rho_{spL})$. It is straightforward to verify that the property $\partial p^{mod}/\partial\rho < 0$ is guaranteed, provided A positive. Moreover, the two spinodal conditions $\partial p^{mod}/\partial\rho(\rho_{spV}) = 0$ and $\partial p^{mod}/\partial\rho(\rho_{spL}) = 0$ are automatically satisfied. The continuity of the pressure and of the free energy at the vapor spinodal density are already enforced through the two integration constants. Such a family of modified pressure functions, Eq. 2, shows a strong sensitivity on the free parameter n (see the right panel of Fig. 1). However, the obtained nucleation rate, left panel of Fig. 1, is almost independent on this parameter, i.e. on the shape of the EoS in the unstable region. Consequently, the cavitation pressure is also unaffected. It is worth noticing that, at variance with the cavitation pressure, the specific expression of f^{mod} enables to partially control the interfacial thickness. In particular, the temperature dependent thickness, commonly defined as the width of the transition layer where the density varies from $\rho_{10} = 0.1\rho_L + 0.9\rho_V$ to $\rho_{90} = 0.9\rho_L + 0.1\rho_V$ (see [2]), reads:

$$\ell_{10-90} = \int_{\rho_{10}}^{\rho_{90}} \sqrt{\frac{\lambda}{\omega(\rho)}}\mathrm{d}\rho\,, \tag{4}$$

with $\omega(\rho) = f_b(\rho) - f_b(\rho_L) - \mu_{sat}(\rho - \rho_L)$. The obtained values are reported in Tab. 1. These two evidences suggest that capturing the exact interfacial thickness is not crucial for the proper evaluation of the cavitation pressure. This issue might play, in principle, an important role from the numerical standpoint, since a water model with an artificially enlarged interface – requiring a coarser numerical mesh, and resulting in a cheaper computational cost – might be able to correctly reproduce the cavitation limit of water.

As a comparison, Tab. 2 reports the thickness values at the different temperatures evaluated with the modified EoS adopted in the main text.

A major effect is observed when changing the spinodal line, as also shown in [2]. Two different versions of the IAPWS EoS are compared in Fig. 2, namely the IAPWS-95 adopted in the main text [1] and the IAPS-82 [3]. Quantitatively, a variation of 3.5% on p_{cav} is measured when the liquid spinodal is varied by 11% (at $T = 50°\mathrm{C}$).

2 Tolman fit

As explained in *Materials and Methods*, an iterative best fitting procedure has been exploited to estimate the Tolman length from the DI surface tension σ^* data. For each temperature

T[°C]	ℓ_{10-90}[nm]
25	1.06554
75	0.996456
125	1.07757
175	1.21998
225	1.43059
275	1.80666
325	2.82201

Table 2: Interface thickness ℓ_{10-90} in the whole range of temperatures analyzed in the main text.

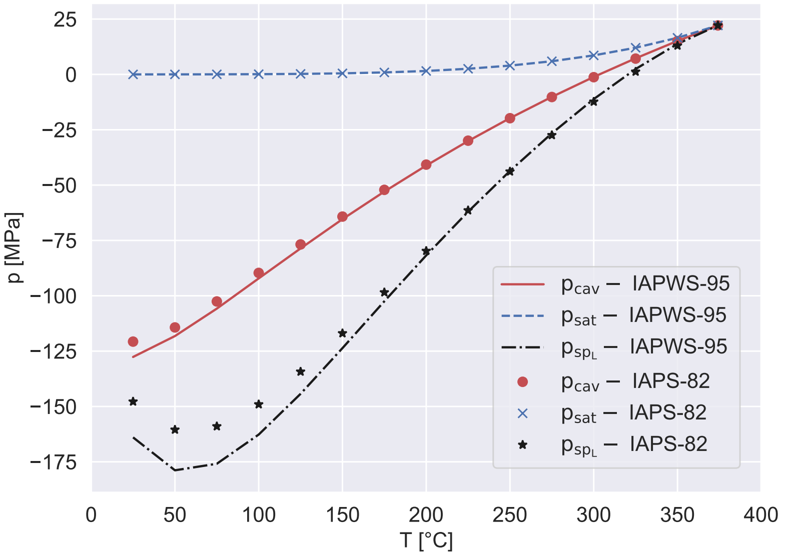

Figure 2: Comparison between the cavitation, saturation and spinodal pressures described by two different versions of the IAWPS EoS. Lines are used for the most recent one, the IAWPS-95 EoS adopted in the main text, while symbols correspond to the older IAPS-82 version.

T, the dataset consists of the set $\{\sigma_i^* = \sigma^*(\mu_{lev}^i, T)\}$, measured at the i-th metastability level $(i = 1, M)$. Iterating over n, at fixed T, the Tolman length δ_n is obtained by using the Tolman law as fitting function $\sigma_i^*(T) = \sigma_0(T)/(1+2\delta_n(T)/R_i^*)$, with $\sigma_0(T)$ the temperature dependent water surface tension of the flat interface provided by [4]. At the n-th iteration level, the fit is obtained by employing the subset $\{\sigma_i^*(T)\}$ with i from $n+1$ to M, thus progressively eliminating from the fit the data at the smallest radii. We employed the L2 norm of the difference between the "corrected"-CNT estimate and the rate measured from the DI model, $|| \log_{10} J_n - \log_{10} J^{DI}|| = (\sum_{i=1}^{M} |\log_{10} J_n(i) - \log_{10} J^{DI}(i)|^2)^{1/2}$, as a target function to be minimized. The fitting error for the different n are reported in Tab. 3, where the optimal value is highlighted in bold.

n	$\delta_n(50°C)$	ϵ	$\delta_n(150°C)$	ϵ	$\delta_n(250°C)$	ϵ	$\delta_n(350°C)$	ϵ
1	0.0879817	12.45	0.156303	48.64	0.194592	38.65	0.34501	22.09
2	0.0809857	7.97	0.115066	27.45	0.144195	22.31	0.277128	14.53
3	0.0765116	6.55	0.0842399	11.97	0.111667	12.15	0.240175	10.81
4	0.0768202	6.58	0.0757209	8.96	0.098805	9.02	0.207386	8.28
5	**0.0758171**	**6.51**	**0.068355**	**8.14**	**0.0888806**	**7.8**	**0.184121**	**7.44**
6	0.0770874	6.62	0.0626723	9.06	0.0815373	7.95	0.167872	7.54
7	0.0756146	6.51	0.0602412	9.81	0.0766019	8.58	0.154278	8.06
8	0.0774596	6.69	0.0591273	10.2	0.0740873	9.04	0.151713	8.2

Table 3: $\epsilon = || \log_{10} J_n - \log_{10} J^{DI}||$. Optimal δ is highlighted in bold.

3 Cavitation pressure sensitivity to the prefactor

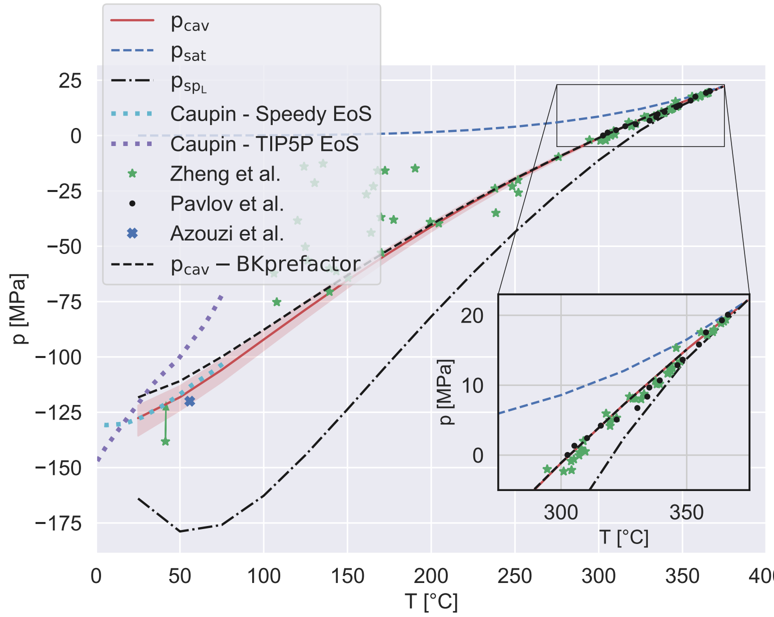

Figure 3: Analysis of the sensitivity of the cavitation pressure on the prefactor in the nucleation rate expression. The Figure compares the value of p_{cav} obtained by employing the Blander&Katz (BK), black dotted line, with the data shown in Fig. 1 of the main text. The effect is comparable with the error band (light red) obtained by varying the experimental $V_l\tau$ parameter.

As discussed in the text, the cavitation pressure is defined as the liquid pressure at which $J(p_{cav}) = \log(2)/(V_l\tau)$, with V_l the system volume and τ the observation time window of the experiment. As a consequence, the nucleation rate plays a key role in the evaluation of the cavitation limit. Both Kramers' theory [5] and kinetics theory [6] provide the nucleation rate being proportional to the exponential of the energy barrier, $J = \Gamma_0 \exp(-\Delta\Omega^*/(k_B T))$, where Γ_0 assumes different expressions. Figure 3 shows the sensitivity of the cavitation pressure on the specific expression of the prefactor. In particular, the one adopted in the main text – derived in [7] – is compared with the classical Blander&Katz expression,

$\Gamma_0 = (\rho_L/m)\sqrt{2\sigma/(m\pi)}$ with m the water molecule mass. The results differ only by few %, highlighting the (expected) major importance of properly capturing the energy barrier.

References

[1] Wolfgang Wagner and Andreas Pruß. The iapws formulation 1995 for the thermodynamic properties of ordinary water substance for general and scientific use. *Journal of physical and chemical reference data*, 31(2):387–535, 2002.

[2] Frédéric Caupin. Liquid-vapor interface, cavitation, and the phase diagram of water. *Physical Review E*, 71(5):051605, 2005.

[3] Joseph Kestin, JV Sengers, B Kamgar-Parsi, and JMH Levelt Sengers. Thermophysical properties of fluid h2o. *Journal of Physical and Chemical Reference Data*, 13(1):175–183, 1984.

[4] T Petrova and RB Dooley. Revised release on surface tension of ordinary water substance. *Proceedings of the International Association for the Properties of Water and Steam, Moscow, Russia*, pages 23–27, 2014.

[5] Peter Hänggi, Peter Talkner, and Michal Borkovec. Reaction-rate theory: fifty years after kramers. *Reviews of modern physics*, 62(2):251, 1990.

[6] Milton Blander and Joseph L Katz. Bubble nucleation in liquids. *AIChE Journal*, 21(5):833–848, 1975.

[7] Georg Menzl, Miguel A Gonzalez, Philipp Geiger, Frédéric Caupin, José LF Abascal, Chantal Valeriani, and Christoph Dellago. Molecular mechanism for cavitation in water under tension. *Proceedings of the National Academy of Sciences*, 113(48):13582–13587, 2016.
